# Yi Qi Qing Re Gao Attenuates Podocyte Injury and Inhibits Vascular Endothelial Growth Factor Overexpression in Puromycin Aminonucleoside Rat Model

**DOI:** 10.1155/2014/375986

**Published:** 2014-05-21

**Authors:** Yongli Zhan, Liping Yang, Yumin Wen, Huijie Liu, Haojun Zhang, Bin Zhu, Wenbing Han, Yanting Gu, Xueyan Sun, Xi Dong, Tingting Zhao, Huixia Ma, Ping Li

**Affiliations:** ^1^Department of Nephrology, Guang'anmen Hospital of China Academy of Traditional Chinese Medical Sciences, No. 5 Beixiange, Xicheng District, Beijing 100053, China; ^2^Beijing University of Chinese Medicine, No. 11 Beisanhuan Donglu, Chaoyang District, Beijing 100029, China; ^3^Department of Pharmacology, Institute of Clinical Medical Sciences, China-Japan Friendship Hospital, No. 2 Yinghua Dongjie, Hepingli, Beijing 100029, China

## Abstract

Proteinuria is the hallmark of chronic kidney disease. Podocyte damage underlies the formation of proteinuria, and vascular endothelial growth factor (VEGF) functions as an autocrine/paracrine regulator. Yi Qi Qing Re Gao (YQQRG) has been used to treat proteinuria for more than two decades. The objective of this study was to investigate the protective effect and possible mechanisms of YQQRG on puromycin aminonucleoside (PAN) rat model. Eighty male Sprague-Dawley rats were randomized into sham group, PAN group, PAN + YQQRG group, and PAN + fosinopril group. Treatments were started 7 days before induction of nephrosis (a single intravenous injection of 40 mg/kg PAN) until day 15. 24 h urinary samples were collected on days 5, 9, and 14. The animals were sacrificed on days 3, 10, and 15, respectively. Blood samples and renal tissues were obtained for detection of biochemical and molecular biological parameters. YQQRG significantly reduced proteinuria, elevated serum albumin, and alleviated renal pathological lesions. YQQRG inhibited VEGF-A, nephrin, podocin, and CD2AP mRNA expression and elevated nephrin, podocin, and CD2AP protein levels starting on day 3. In conclusion, YQQRG attenuates podocyte injury in the rat PAN model through downregulation of VEGF-A and restoration of nephrin, podocin, and CD2AP protein expression.

## 1. Introduction

Clinical and experimental studies indicate that proteinuria is the major risk factor for CKD progression and glomerular sclerosis and also a marker of increased cardiovascular morbidity and mortality [1]. The glomerular filtration of albumin is followed by receptor-mediated endocytosis of the proximal tubular cells, so both the glomerular filtration barrier (GFB) and tubular dysfunction are involved in the initiation of proteinuria, leading to increased excretion of albumin in the urine. Loss of selectivity of GFB to protein filtration, however, is the common final pathway for proteinuric glomerulopathies, independent of the underlying causes. The GFB is composed of the fenestrated endothelium, the basement membrane, the podocyte, and its slit diaphragm [2]. Increasing evidence suggests that podocytes are the essential determinant of GFB function. Podocytes are highly differentiated cells with finger-like foot processes connecting its adjacent compartments with slit-diaphragm (SD) molecules. The SD is the size-selective permeability barrier for the passage of plasma proteins across the GFB. When podocytes are injured, their cytoskeletal structure and intercellular junctions are damaged, resulting in morphological change called foot process effacement, which leads to proteinuria. Further manifestations are podocyte detachment from the GBM and glomerular sclerosis [3, 4]. Up to now, the molecular basis of SD structure has been illustrated. Nephrin, encoded by the NPSH1 gene, is a transmembrane glycoprotein, belonging to the immunoglobulin superfamily that functions as the structural backbone of SD. Nephrin consists of eight extracellular Ig domains, followed by a fibronectin type III-like domain, a short transmembrane region, and a cytoplasmic C-terminus. The cysteine in the fibronectin type III-like domain can form disulfide bond with the adjacent nephrin molecule to form a zipper-like structure of SD [5]. Mutations of the nephrin gene results in congenital nephrotic syndrome of the Finnish type (CNF), characterized by massive proteinuria, podocyte foot process effacement, and absence of the slit diaphragm [6, 7]. Nephrin also functions as an intracellular signaling scaffold, which interacts with the adapter protein CD2AP and podocin. The N-terminus of CD2AP contains a Src homology 3 (SH3) domain and functions as an adaptin. CD2AP interacts with podocin and nephrin via its C-terminus, locating its anchor within lipid rafts in order to preserve the functions of the cytoskeleton and SD. Damage to CD2AP directly damages the podocyte cytoskeleton, leading to deformation and disappearance of podocyte and massive proteinuria. In addition, CD2AP can interact with various signaling molecules via its SH3 region and participate in cytoskeleton assembly [7–9]. Yaddanapudi et al. substantiated that CD2AP regulates TGF-*β*1-dependent translocation of dentrin from the SD to nucleus and functions as a gatekeeper for reorganization of the podocyte microfilament system and consequent proteinuria [10]. Podocin is encoded by NPSH2 gene and is a new member of the stomatin family of lipid raft-associated proteins. Podocin functions as a supporting protein in the maintenance of the podocyte process and integrity of SD [11]. Other proteins, including Neph1, FAT-1 [12], ZO-1 [13], and podocalyxin [14], are noted in animal mutant models, and mutations of any of these proteins result in massive proteinuria and early postnatal death.

Vascular endothelial growth factor-A (VEGF-A) is an angiogenesis and endothelial survival factor. It is also a critical “cross-talk” protein among the three components of GFB [15]. Podocytes are the major site of VEGF expression in the glomeruli. VEGF-A binds to its receptors VEGFR1 (Flt-1), VEGFR2 (Flk-1), and coreceptors neuropilin 1 and 2. There are three isoforms of VEGF-A that podocytes synthesize: VEGF121, 165, and 189 by alternative mRNA splicing [16]. Sison et al. demonstrated that VEGF regulates slit diaphragm signaling through VEGFR2-nephrin cross-talk [17]. VEGF gain of function downregulates nephrin expression and distorts podocyte morphology, which concurs with the foot process effacement and proteinuria [18–20]. The autocrine VEGF signaling system in podocytes also regulates SD proteins by inducing podocin upregulation and increasing its interaction with CD2AP* in vitro* [16].

YQQRG has been used in Guang'anmen Hospital to treat CKD for more than two decades, previous study demonstrated that YQQRG reduces proteinuria, elevates serum albumin, and decreases serum cholesterol in patients with chronic nephritis [21]. Animal experiments have shown that the formula inhibits extracellular matrix accumulation in adriamycin nephrosis [22]. However, the mechanism of YQQRG in the treatment of proteinuria remains unknown. Since podocyte injury has been found to be the main pathogenesis of proteinuria, our study investigated whether YQQRG possesses a regulatory effect on podocytopathy and VEGF expression.

## 2. Materials and Methods

### 2.1. Drugs and Reagents

**Figure 1:**
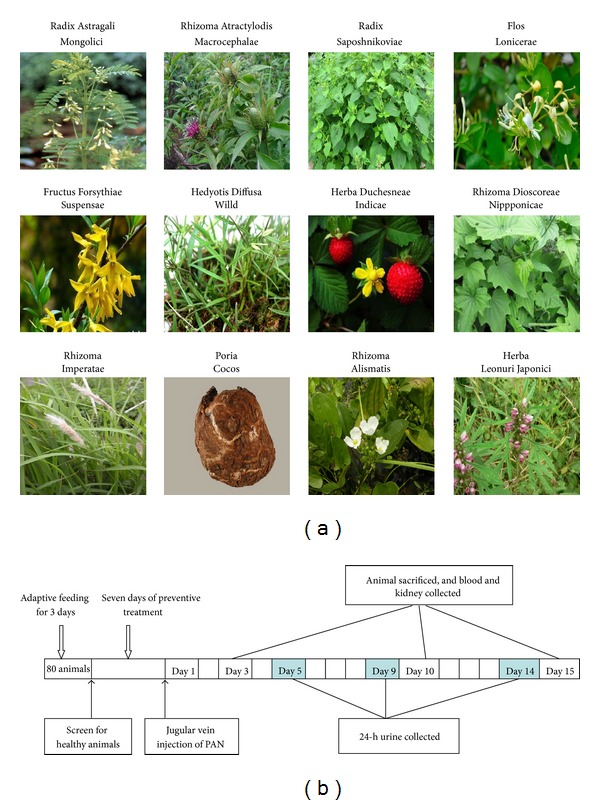
Composition of YQQRG and study design. (a) Plants of the components of YQQRG. (b) Time plot of study design.

YQQRG was manufactured by Guang'anmen Hospital, (certificate number: 98 Beijing Health and Drug number 058). The formula includes Radix Astragali Mongolici (*huang qi*) 72 g, Rhizoma Atractylodis Macrocephalae* (bai zhu*) 54 g, Radix Saposhnikoviae (*fang feng*) 36 g, Flos Lonicerae (*jin yin hua*) 72 g, Fructus Forsythiae Suspensae (*lian qiao*) 72 g, Herba Duchesneae Indicae (*she mei*) 54 g, Hedyotis Diffusa Willd (*bai hua she she cao*) 180 g, Poria Cocos (*fu ling*) 72 g, Rhizoma Alismatis (*ze xie*) 125 g, Herba Leonuri Japonici (*yi mu cao*) 180 g, Rhizoma Imperatae (*bai mao gen*) 180 g, and Rhizoma Dioscoreae Nipponica (c*huan shan long*) 70 g. The herbal medicinals were prepared through a process of decoction, concentration, purification by ethanol precipitation, and sterilization. The final standardized product was 4.5 g crude medicinal per milliliter (Figure 1(a)). Fosinopril sodium tablets (10 mg/tablet) were purchased from Bristol-Myers Squibb Shanghai (Lot number: H19980197).

PAN and podocin monoclonal antibody were purchased from Sigma-Aldrich (St. Louis, MO, USA); nephrin monoclonal antibody was purchased from Santa Cruz Biotech (Santa Cruz, CA, USA); CD2AP and VEGF-A monoclonal antibodies were purchased from Abcam (Cambridge, UK); *β*-actin monoclonal antibody, goat anti-mouse IgG, and goat anti-rabbit IgG antibodies were purchased from Zhongshan Jinqiao (Shanghai, China); immunohistochemical kit was purchased from Gene Technology Company (Shanghai, China). Other chemicals and reagents were of analytical grade.

### 2.2. Animals and Experimental Design

Eighty male Sprague-Dawley rats, weighing 90–100 g were purchased from Beijing HFK Bio-Technology Co. Ltd. (Beijing, China, Certificate No. SCXK-(Jing) 2009-0007). All animals were housed at 22 ± 3°C and 50 ± 10% humidity, with 12 h light/dark cycle, and were given free access to tap water and standard chew. This study was approved by the Ethics Committee of China-Japan Friendship Hospital and performed in accordance with the Guiding Principles for the Care and Use of Laboratory Animals (No. 2010-A10).

Animals were randomized to four groups after 1 week of adaptive feeding: sham group, PAN group, PAN + YQQRG treatment group (YQQRG, 4 g/kg, 14 times human dosage), and PAN + fosinopril treatment group (1.667 mg/kg, 10 times human dosage). Animals in PAN and both treatment groups were anesthetized with an intraperitoneal injection of 10% chloral hydrate and then received a single intravenous injection of 40 mg/kg PAN through a cannula in the right internal jugular vein within 5 min. Animals in the sham group were injected with equal volume of saline. All drugs were dissolved in distilled water and given by gastric gavage once daily 7 days before induction of nephrosis until day 15. On days 5, 9, and 14 after PAN injection, the animals were housed individually in metabolic cages, deprived of food and with free access to water. Collections of 24-hour urine were obtained. Total urinary protein was measured by the Bradford method. Six rats from each group were anesthetized with an intraperitoneal injection of 10% chloral hydrate and sacrificed on days 3, 10, and 15 after PAN injection (Figure 1(b)). Blood samples were taken from the abdominal aorta and serum was separated for measurement of alanine transarninase (ALT), aspartate aminotransferase (AST), creatinine (Cre), blood urea nitrogen (BUN), total protein (TP), albumin (Alb), total cholesterol (TC), triglycerides (TG), high density lipoprotein (HDL), and low density lipoprotein (LDL). Samples were processed using an automatic biochemistry analyzer (RA-1000 CS; Technicon, USA).

### 2.3. Histological Examination of the Kidney

Sections of kidney were fixed in 10% phosphate buffered formalin solution for 24 h, embedded in paraffin, sectioned at 3 *μ*m slices, and stained with periodic acid-schiff stain. Slices were examined microscopically and photographed (BX-51 Research Microscope system and DP70 Image Acquisition System; Olympus, Japan). For electron microscopy examination, small samples of the right kidney were removed and cut into 1 mm^3^, fixed in 2.5% glutaraldehyde, dehydrated with gradient acetone, embedded with ethoxyline resin Epon 812, sliced by ultrathin slicing machine, and examined by transmission electron microscopy (H-600, Hitachi, Japan).

### 2.4. Immunohistochemistry

Immunohistochemistry stain of nephrin was conducted following the manufacturer's instructions. Briefly, slices of 3 *μ*m kidney sections were put into 60°C oven for 2 hours. After gradient deparaffinization and rehydration, the slices were immersed in microwave heated 0.01 mol/L citrate buffer at 100°C for retrieval of antigen sites. Endogenous peroxidase activity was quenched by incubation with 3% H_2_O_2_. The slices were incubated with nephrin antibody (1 : 100) at 4°C overnight. After three 5-min washes with PBS, each section was incubated with the secondary antibody at 37°C for 30 min. The visualization of slices was developed with diaminobenzidine (Dako Corp., Carpinteria, CA) and was counterstained with hematoxylin. The semiquantitative analysis of nephrin was determined using the Image-Pro Plus 6.0 image analysis software (Media Cybernetics, Warrendale, PA, USA). Briefly, ten random fields of glomerular under an upright microscope at 400x magnification were outlined and positive staining patterns were identified. The percentage of positive stained area occupying the selected glomeruli was calculated. The analysis was performed with blindness of groups.

### 2.5. Real-Time Polymerase Chain Reaction (RT-PCR)

Total RNA of renal cortex was isolated using total RNA Kit (R6934, Omega Bio-tech Inc., GA, USA) following the manufacturer's instructions. cDNA was synthesized in cDNA Synthesis Kit (K1622, Fermentas International Inc., Canada) according to the manufacturer's instructions. Each PCR was performed in triplicate in a final volume of 20 *μ*L solution: 10 *μ*L of SYBR Green dye, 1 *μ*L of diluted cDNA products, 0.2 *μ*L of each paired primer, and 8.6 *μ*L deionized water. Protocols were as follows: initial denaturation for 10 min at 95°C, followed by 30 cycles (for nephrin, podocin, and VEGF-A) or 32 cycles (for CD2AP and Cyclophilin-B) denaturation for 15 s at 95°C, and extension for 30 s at 60°C (for VEGF-A and Cyclophilin-B) or 58°C (for nephrin, podocin, and CD2AP). The last cycle for dissociation of SYBR Green probe was 15 s at 95°C, 30 s at 60°C, and 15 s at 95°C. Cyclophilin-B was used as a house-keeping gene as previously reported [23]. Threshold cycle (*C*_*T*_) values of target genes were measured and normalized to that of Cyclophilin-B and expressed as a relative ratio. The specific primer sequences were nephrin: forward 5′-ATGGGCGCTAAGAGAGTCAC-3′ and reverse 5′-CGCAGTCAGGTTTTCAGACA-3′. Podocin: forward 5′-TCTTGTCCTCTCCTCCCTGA-3′ and reverse 5′-AGACGGAGGTCAACCTTGTG-3′. CD2AP: forward 5′-GCTGGTGGAAAGGTGAACTG-3′ and reverse 5′-CATCTCTGTCTTCCGCCTTC-3′. VEGF-A: forward 5′-ACTGGACCCTGGCTTTACTGC-3′ and reverse 5′-TTGGTGAGGTTTGATCCGCATG-3′. Cyclophilin-B: forward 5′-CCATCGTGTCATCAAGGACTTCAT-3′ and reverse 5′-TTGCCGTCTAGCCAGGAGGTCT-3′. The Δ*C*_*T*_ value was determined by subtracting the Cyclophilin-B *C*_*T*_ value of each sample from its respective target *C*_*T*_. Fold changes of the target gene were equivalent to 2^−ΔΔ*C*_*T*_^ [24].

### 2.6. Western Blot Analysis

Renal cortices were homogenated with lysis buffer and 1x protease inhibitor cocktail in a tissue homogenator (Roche Diagnostics, Indianapolis, IN, USA) and then centrifuged. The supernatant was extracted to measure the concentration of protein. The protein samples were then added in 5x SDS-PAGE loading buffer and were heated at 100°C for 10 min to be denatured. Proteins (80 *μ*g) were separated by 12% SDS-polyacrylamide gel electrophoresis (PAGE) and transferred to an immunoblot polyvinylidene fluoride (PVDF) membrane (Bio-Rad, Hercules, CA, USA). After blocking in PBS/Tween (0.1%) with 5% skim milk for 2 hours, the membrane was incubated with primary antibodies (nephrin antibody 1 : 500, podocin antibody 1 : 2000, and CD2AP antibody 1 : 1000) overnight at 4°C. Target bands were detected using a horseradish peroxidase-conjugated secondary antibody and developed using Pierce enhanced chemiluminescence (ECL) (ThermoScientific, Fisher Scientific, Pittsburgh, PA, USA). Band intensity of nephrin, podocin, CD2AP, and VEGF-A was normalized to that of *β*-actin and expressed as a relative ratio. Semiquantifications were performed using Image J program (National Institutes of Health, Bethesda, MD, USA).

### 2.7. Statistical Analysis

Data were presented as means ± standard deviation (SD) unless otherwise stated. Comparison of measurement data between groups were evaluated by the analysis of variance (ANOVA), followed by Student-Newman-Keuls (SNK) test. *P* values <0.05 were regarded as statistically significant. All statistical analyses were performed by SPSS 17.0.

## 3. Results

### 3.1. YQQRG Attenuated Urinary Protein Excretion in PAN Rats

**Figure 2:**
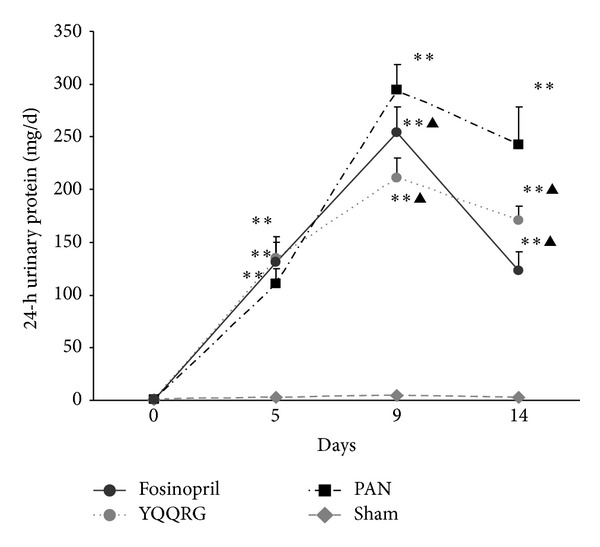
YQQRG attenuated 24 h urinary protein in PAN induced nephrosis rats. 24 h urinary protein elevated significantly after PAN injection. YQQRG attenuated 24 h urinary protein excretion of PAN rats on days 9 and 14, an effect equivalent to that of the fosinopril. (Compared with sham group: *P < 0.05, **P < 0.01, compared with PAN group: ^▲^ *P* < 0.05, ^▲▲^ *P* < 0.01.)

The 24 h urinary protein level of each group was within normal range before injection of PAN. Urinary protein increased significantly at day 5, peaked at day 9, and slightly decreased at day 14 after PAN injection. There was significant reduction of urinary protein level at days 9 and 14 in YQQRG treated group, an effect equivalent to that of fosinopril (Figure 2).

### 3.2. YQQRG Attenuated Serum Alb Levels and Regulated Lipid Disorder without Apparent Liver and Kidney Adverse Effects

There was no significant difference among groups at day 3 for each of the biochemical parameters. There were significant decrease of TP, Alb, and elevation of TC, TG, HDL, and LDL levels of the PAN group compared with that of the sham group at days 10 and 15 (*P* < 0.05). YQQRG could significantly reduce TG at day 10, equivalent to that of the fosinopril. At day 15, YQQRG increased plasma levels of TP and Alb and reduced TC, TG, and LDL levels (*P* < 0.05 or *P* < 0.01), equivalent to that of the fosinopril. No obvious differences were observed for ALT, AST, BUN, and Cre among each group at every time point (Table 1).

### 3.3. YQQRG Alleviated Kidney Morphological Lesions in PAN Rats

**Figure 3:**
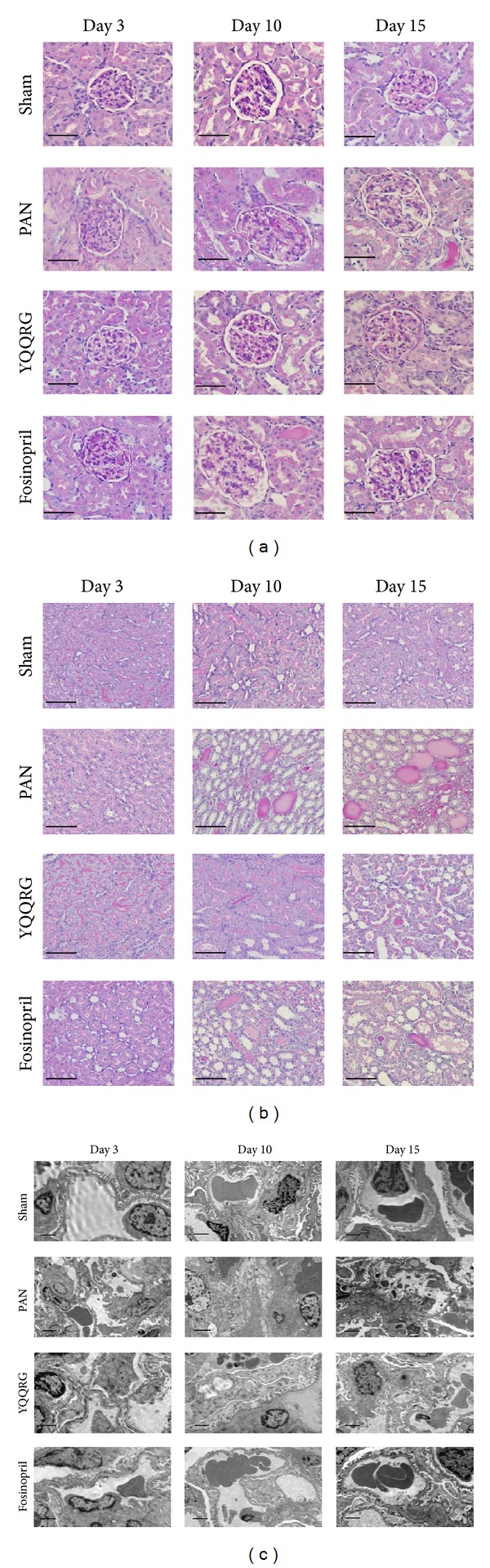
YQQRG alleviated kidney morphologic lesions in PAN rats. (a) Rat in PAN group exhibited glomerular hypertrophy on days 10 and 15; no other pathological lesions were seen (PAS, magnification ×400, scale bar = 50 *μ*m). (b) Extension of tubules with protein casts; interstitial inflammatory cell infiltration was obvious in the PAN group on days 10 and 15. YQQRG attenuated such lesions (PAS, magnification ×400, scale bar = 50 *μ*m). (c) Diffuse effacement of podocyte foot process, mitochondria swelling, and disarrangement of microfilaments of podocytes occurred on day 3 and worsened on day 10. However, there was a trend of spontaneous attenuation on day 15. YQQRG protected against podocyte injury (magnification ×10000, scale bar = 2 *μ*m).

Pathological results revealed that there were no apparent pathological changes for both glomeruli and tubules at day 3. Together with elevated 24 h urinary protein, 10 and 15 days after PAN injection, there was apparent glomerular hypertrophy though no other pathologic lesions were seen in this study (Figure 3(a)). Typical tubular-interstitial lesions included protein casts, tubular protein reabsorption droplets, and interstitial inflammatory cells infiltration. Treatment with YQQRG and fosinopril appeared to alleviate such pathologic changes with similar degrees (Figure 3(b)).

Glomerular ultrastructure studies revealed obvious diffuse effacement of podocyte foot process, swelling of mitochondria, and disarrangement of microfilaments of the podocytes on day 3, a phenomenon that occurred before appearance of massive proteinuria. These lesions were worsened by day 10, with vacuolar degeneration of the podocyte cell body and universal effacement of foot process. However, on day 15, there was a trend of spontaneous attenuation, with less severe vacuolar degeneration and foot process effacement. Treatment with YQQRG significantly alleviated podocyte damage with significant reduction in foot process fusion and mitochondria swelling, equivalent to the effects of fosinopril (Figure 3(c)).

### 3.4. YQQRG Restored Nephrin, Podocin, and CD2AP Protein Expression and Downregulated mRNA Expression

**Figure 4:**
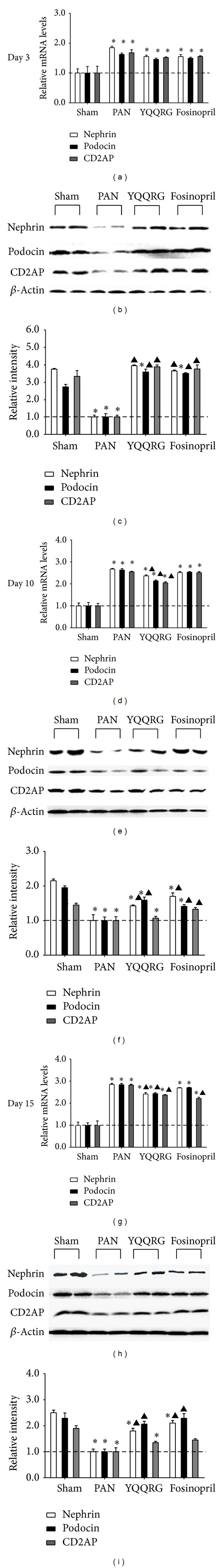
YQQRG downregulated nephrin, podocin, and CD2AP mRNA expression and restored their protein expression. Nephrin podocin and CD2AP mRNA expression was upregulated about 2-fold in PAN group compared with sham group on day 3 (a) and upregulated about 3-fold on days 10 and 15 (d and g). Treatment with YQQRG significantly inhibited the mRNA expression of these genes on days 10 and 15 (a, d, and g). Western blot revealed that compared with sham group, nephrin, podocin, and CD2AP expression in the PAN group was significantly decreased starting on day 3 (*P* < 0.05), and treatment with YQQRG restored podocin and nephrin expression on days 3, 10, and 15, however only on day 3 for CD2AP (*P* < 0.05) (b, c, e, f, h, i). (Compared with sham group: *P < 0.05, **P < 0.01, compared with PAN group: ^▲^ *P* < 0.05, ^▲▲^ *P* < 0.01.)

**Figure 5:**
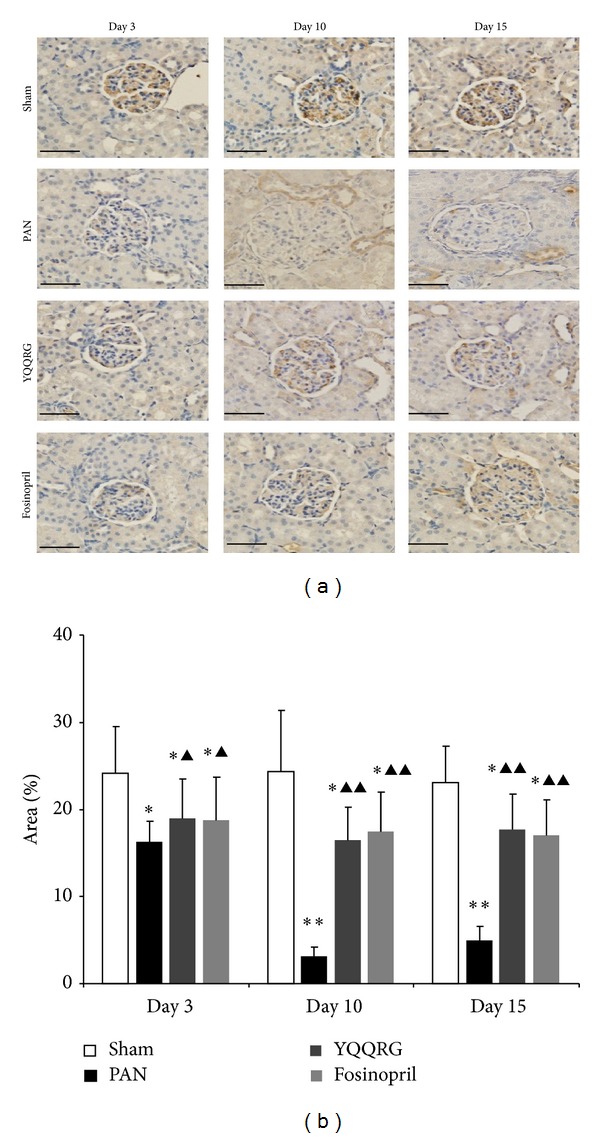
YQQRG restored podocyte nephrin expression. Immunohistochemistry and semiquantitative analysis revealed that nephrin expression was decreased at day 3 and further declined at days 10 and 15. YQQRG could restore nephrin expression at every time point, equivalent to that of fosinopril (magnification ×400, scale bar = 50 *μ*m).

To validate the protective effect of YQQRG on podocyte injury, the expressions of podocyte slit-diaphragm (SD) molecules nephrin, podocin, and CD2AP at mRNA and protein levels of renal cortex samples were detected by real-time PCR and western blot analysis. Localization of nephrin was detected by immunohistochemistry. Real-time PCR revealed that mRNA expressions of nephrin, podocin, and CD2AP were upregulated in PAN group compared with the sham group as of day 3. Treatment with YQQRG could significantly inhibit the mRNA expression of these genes on days 10 and 15 (Figures 4(a), 4(d), and 4(g)). Western blot revealed that compared with sham group, nephrin, podocin, and CD2AP expression in the PAN group was significantly decreased starting on day 3 (*P* < 0.05), and treatment with YQQRG restored podocin and nephrin expression on days 3, 10, and 15, however only on day 3 for CD2AP (*P* < 0.05) (Figures 4(b), 4(c), 4(e), 4(f), 4(h), and 4(i)). Nephrin expression detected by immunohistochemistry and data analysis revealed that podocyte nephrin expression was decreased as of day 3, and treatment with YQQRG could restore nephrin expression at every time point (Figure 5).

### 3.5. YQQRG Attenuated Podocyte Injury through Inhibition of VEGF-A Overexpression

**Figure 6:**
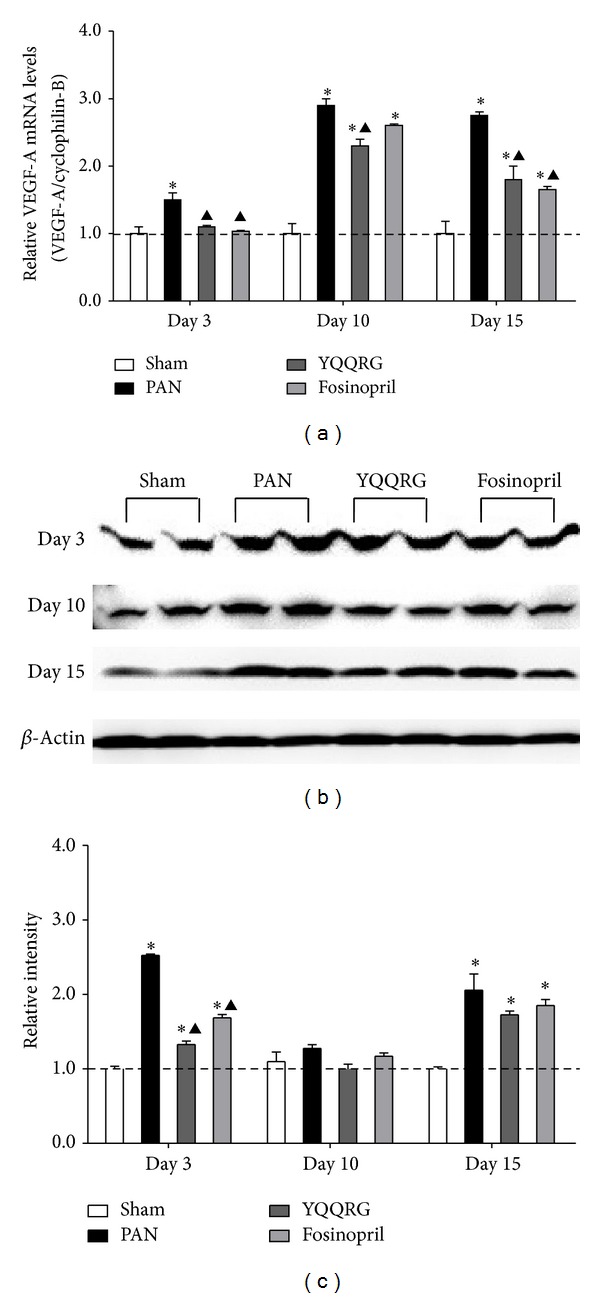
YQQRG decreased VEGF-A expression. VEGF-A level was increased early on day 3 and continued to day 15. Treatment with YQQRG attenuated VEGF expression. (Compared with sham group: *P < 0.05, **P < 0.01, compared with PAN group: ^▲^ *P* < 0.05, ^▲▲^ *P* < 0.01.)

To obtain insights into the regulatory mechanisms of YQQRG on SD molecule expression, we further investigated the effects of YQQRG on expression of VEGF-A. The mRNA expression of VEGF-A was significantly increased as of day 3, and YQQRG was able to inhibit its expression (Figure 6(a)). Western blot analysis revealed that VEGF-A expression was increased on day 3 and continued increasing until day 15. Treatment with YQQRG decreased VEGF-A levels on days 3, 10, and 15, an effect equivalent to that of fosinopril (Figures 6(b) and 6(c)).

## 4. Discussion

Proteinuria has been demonstrated to be related to adverse outcomes in patients with CKD. A high level of proteinuria increases the risks of myocardial infarction, kidney failure, and mortality [1]. Treatments for proteinuria currently include three kinds of therapeutic agents: glucocorticoids, cytotoxic drugs, and angiotensin-converting enzyme inhibitors (ACEIs) or angiotensin II receptor blockers (ARBs). Despite disease-specific functions of glucocorticoids and cytotoxic drugs, the most widely applied therapy for proteinuria is ACEI/ARBs. Clinical investigations have impressively demonstrated that ACEI/ARBs, used alone or combined, can relieve the severity of proteinuria, slow progressive loss of kidney function, independent of their blood pressure lowering effect [25]. However, ACEI/ARBs are not curative and most patients still have renal morbidity and mortality. Exploration for new therapies and their mechanisms, including traditional Chinese medicine, is a constant topic in this field. YQQRG is a widely used formula in Guang'anmen Hospital, with demonstrated antiproteinuric and antisclerotic effects [21, 22]. The current study investigates the effect of YQQRG on podocyte injury and VEGF expression in the kidney tissue of PAN model, with fosinopril, an ACEI, as control for the treatment of proteinuria.

PAN model is a classic model to study the mechanism of proteinuria and podocytopathy, in which PAN specifically injures the GFB and manifests as podocyte foot process effacement, depletion of podocyte number, and direct damage to the negative-charged barrier [26]. Podocyte apoptosis is an early and important manifestation in PAN nephrosis, with apparent cell membrane inversion and mitochondrial membrane potential depolarization [27]. Estrogen receptor *α* and nestin are demonstrated to protect podocyte from apoptosis [27, 28]. Shibata et al. reported that 12 h after a single intravenous injection of PAN (10 mg/100 g), there was reduced staining of nephrin detected by immunofluorescence and significant decrease of nephrin mRNA expression in the glomeruli [29]. Similar results were seen 5 days after PAN administration at the same dosage [30]. However, there are discrepancies regarding mRNA expression of nephrin and podocin. Subcutaneous injection of PAN (120 mg/kg) induced decreased protein expression of nephrin and podocin from days 3 to 5 after injection, a time that albuminuria begins to develop. However, nephrin mRNA level was significantly enhanced on days 2 and 3 after PAN injection, normalized on day, 4 and decreased on day 5. Podocin mRNA level was not significantly changed from 1 to 5 days after PAN injection [31]. Research by Han et al. revealed that mRNA expression of nephrin and podocin was significantly lower on day 1 and recovered (for nephrin) or increased (for podocin) on day 28 [32]. Discrepancies between these studies are difficult to explain and may be due to different administration of PAN, animal strain, or different time points of observation. In our study, we dynamically observed the 24 h urinary protein, pathological changes, and alterations of nephrin, podocin, and CD2AP expression in the PAN model. 24 h urinary protein increased after PAN injection and peaked at 10 days. Treatment with YQQRG significantly decreased urinary protein and elevated serum albumin. Ultrastructural changes occurred prior to massive proteinuria, including diffuse podocyte foot process effacement, swelling of the mitochondria, and disarrangement of microfilaments. YQQRG attenuated these pathological changes, exhibiting a protective effect on GFB structure, an effect equivalent to that of fosinopril.

In order to study the molecular mechanism of YQQRG on GFB function, we further studied the SD related molecules nephrin, podocin, and CD2AP. The mRNA expressions of nephrin, CD2AP, and podocin were upregulated in the PAN group compared with the sham group as of day 3. Treatment with YQQRG significantly inhibited mRNA expression of these genes on days 10 and 15. However, the protein levels of nephrin, podocin, and CD2AP were decreased early on day 3, and treatment restored their expression, a phenomenon that seems contradictory. A possible explanation is that the alterations of SD molecules in the PAN model are at the translation stage rather than the transcription stage. In passive Heymann nephritis, a discrepancy of nephrin mRNA upregulation and decrease in protein was also detected, due to suppressed translation of nephrin [33]. Human nephrin has 10 potential N-glycosylation sites in its amino acid sequence [34]. It has been reported that N-glycosylation of nephrin plays a crucial role in molecular folding and is critical for plasma membrane localization. In PAN nephrosis, non- or subglycosylated nephrin undergoes unfolded protein response (UPR), and nonglycosylated and incorrectly folded nephrin is retained in the endoplasmic reticulum and is subsequently transported to the cytoplasm for ubiquitination and degradation by proteasomes [19, 35]. The upregulation of nephrin mRNA may be a negative feedback reaction in the defect of posttranslational modification.

VEGF-A is a pleiotropic growth factor essential for endothelial cell differentiation, survival, proliferation, and migration. Podocytes are the major source of VEGF-A in the glomeruli. VEGF-A acts as chemoattractant for endothelial cells, guiding their migration towards developing into nephron during kidney organogenesis [36]. VEGF-A is essential for maintenance of the fenestrated phenotype for glomerular endothelial cells. VEGF-A also interacts with podocyte slit-diaphragm molecules to regulate the integrity of the GFB. Studies have shown that patients who underwent chemotherapy with anti-VEGF or receptor tyrosine kinase inhibitors experienced proteinuria, podocyturia, hypertension, and glomerular thrombotic microangiopathy, indicating damage to the GFB [37–39]. In cultured podocytes, VEGF-A induces podocin upregulation, increases podocin-CD2AP interaction, and downregulates and stimulates nephrin phosphorylation in a dose-dependent manner [18]. Induction of podocyte VEGF-A overexpression causes reversible nephrin downregulation and phosphorylation, associated with foot process effacement and proteinuria [20, 38]. Podocyte derived VEGF functions as an important “cross-talk” protein among the three components of GFB in developing and mature glomeruli. An early study by Fan et al. found a downregulation of VEGF and its receptors in the PAN model [40]. However, increasing evidence confirms that in PAN model, as well as in passive Heymann nephrosis and diabetic nephropathy, upregulation of VEGF and its receptors VEGFR1 and VGFR2 is correlated with severity of proteinuria [41, 42]. In human biopsy specimens, VEGF and its receptor expression as well as VEGF-VEGFR2 complex, without any exception, were increased in the podocyte, indicating an autocrine loop in diseased podocytes [43]. PAN model treated with anti-VEGF serum for the first 5 days of induction of PAN nephrosis exhibited no effect on proteinuria and glomerular filtration rate, a result consistent with VEGF165 aptamer treatment for the PAN model [42, 44]. Experiments using* in vivo* and* in vitro* transgenic models demonstrated that podocyte derived VEGF has a self-protective autocrine function against PAN induced podocyte injury [45]. All these results indicate that VEGF-A and VEGFR expressions probably impact proteinuria in a less direct way, the altered VEGF signaling may be a protective response. In our study, VEGF-A expression was increased on day 3 after PAN injection, simultaneous with nephrin downregulation and podocyte damage. A possible mechanism may be that the direct podocyte toxin of PAN caused suppression of SD molecules. Subsequently, podocyte enhanced VEGF-A expression in a self-protective way. However, VEGF-A overexpression exacerbated podocyte injury and caused further damage. There were also enhanced expressions of both VEGFR1 and VEGFR2 that we were unable to identify the specific source of (data not shown). Thus, YQQRG appears to restore SD function through inhibition of VEGF and upregulation of nephrin, podocin, and CD2AP.

YQQRG is comprised of 12 active constituents. Astragalin (Radix Astragali Mongolici) has been demonstrated to decrease the overexpression of VEGF induced by high glucose in Müller cells [46]. Astragalus saponins have also been found to inhibit human gastric adenocarcinoma cell proliferation and invasion by downregulation of the proangiogenic protein VEGF as well as the metastatic proteins metalloproteinase-2 (MMP-2) and MMP-9 [47]. In the complement membranous attack complex induced podocyte injury model, astragaloside IV (AS-IV) restores podocyte morphology and cytoskeleton loss in a dose- and time-dependent manner. Furthermore, AS-IV was able to reduce phosphorylation of JNK and ERK1/2 induced by complement membranous attack complex [48]. In streptozotocin induced rat diabetic nephropathy model, AS-IV ameliorated albuminuria, renal histopathology, and podocyte foot process effacement and inhibited kidney expression and serum levels of nuclear factors-*κ*B (NF-*κ*B), tumor necrosis factor-*α* (TNF-*α*), monocyte chemotactic protein-1 (MCP-1), and intercellular cell adhesion molecule-1 (ICAM-1) [49]. Astragalin has been reported to inhibit angiotensin converting enzyme (ACE) activity* in vitro* [50]. Nevertheless, there are reports that renal local angiotensin II (Ang II) is not affected by combination of Astragalus and Angelica (AA). The AA retarded the progression of renal fibrosis and renal function deterioration, which is similar to the effect of ACEI. These renoprotective effects were not via inhibitory renal Ang II, whereas they were associated with suppression of TGF-*β*1 and osteopontin overexpression, reduction of both infiltration of monocyte/macrophage, and activation of renal intrinsic cells [51]. The actions of a single component and mixture of a formula are quite different. The actions of formula may be related to multiple pathways and mechanisms. Ethanol extract of honeysuckle flower (Flos Lonicerae) ameliorates kidney injury in diabetic rats, reduces macrophage and T cell infiltration, and attenuates expression of proinflammatory cytokines, by downregulation of p38-mitogen-activated protein kinase in diabetic rats [52]. Hedyotis Diffusa Willd (HDW) is a herbal medicinal widely used in the treatment of cancer in China. Recent studies indicate that ethanol extract of HDW inhibits the proliferation, migration, and tube formation of human umbilical vein endothelial cells in a dose- and time-dependent manner [53]. In the colorectal cancer mouse xenograft model, ethanol extract of HDW was found to inhibit sonic hedgehog signaling pathway dependent VEGF-A and VEGFR2 expression [54]. Japanese dioscorea rhizome has been demonstrated to have immune regulatory, antiviral, and antibacterial effects. Its active components are protodioscin and dioscin. Protodioscin was reported to have antihyperlipidemic effect [55]. In lipopolysaccharide-stimulated rat mesangial cells, dioscin inhibited mesangial cell proliferation and downregulated expressions of Wnt4, *β*-catenin, and TGF-*β*1 in a dose- and time-dependent manner [56]. The renal protective effects of YQQRG in PAN rats may be due to the aforementioned modes of the active constituents in the formula.

In conclusion, our data suggest that YQQRG may possess renal protective effect in the proteinuric state via restoration of renal glomerular filtration barrier and by inhibition of VEGF expression. These results indicate that YQQRG is an attractive and suitable alternative therapy for treating proteinuria. Further mechanism studies are being undertaken.

## Figures and Tables

**Table 1:** YQQRG elevated serum Alb levels and regulated lipid disorder.

	Time points (day)	Sham	PAN	YQQRG	Fosinopril
ALT (U/L)	3	43.67 ± 3.21	39.33 ± 2.31	32.75 ± 2.75	39.5 ± 2.06
	10	49.6 ± 7.02	43.67 ± 6.41	47.2 ± 9.58	43.0 ± 3.0
	15	42.0 ± 8.85	32.38 ± 7.17	32.33 ± 3.88	39.0 ± 5.14

ASL (U/L)	3	130.2 ± 8.1	119.98 ± 5.19	103.8 ± 7.21	119.67 ± 5.94
	10	100.94 ± 9.15	73.54 ± 7.42	73.43 ± 18.89	71.74 ± 19.83
	15	155.28 ± 27.54	96.71 ± 22.07	122.12 ± 19.39	116.58 ± 16.90

TP (mmol/L)	3	50.6 ± 5.26	49.53 ± 5.0	50.88 ± 6.57	46.98 ± 5.43
	10	49.98 ± 2.44	44.3 ± 3.50*	46.96 ± 3.57	43.02 ± 2.47*
	15	59.61 ± 9.68	49.44 ± 3.79*	53.3 ± 7.32	52.33 ± 2.06*

Alb (g/L)	3	34.03 ± 3.32	31.73 ± 2.68	32.88 ± 3.22	30.73 ± 2.31
	10	32.4 ± 1.42	23.38 ± 2.39*	24.87 ± 3.40*	24.02 ± 3.05*
	15	36.35 ± 4.35	27.06 ± 3.51*	29.16 ± 3.20^∗▲^	30.06 ± 2.06^∗▲^

BUN (mmol/L)	3	10.18 ± 1.77	10.03 ± 1.59	9.6 ± 1.38	7.48 ± 1.03
	10	5.82 ± 1.57	7.85 ± 0.66	6.73 ± 2.15	5.77 ± 1.40
	15	5.71 ± 1.40	6.33 ± 1.31	6.18 ± 0.73	7.14 ± 1.48

Cre (*μ*mol/L)	3	33 ± 7.94	27.67 ± 5.13	27.0 ± 5.6	16.67 ± 0.47
	10	25.6 ± 1.14	25.6 ± 3.91	26.33 ± 2.89	19.33 ± 1.53
	15	31.17 ± 3.82	27.14 ± 4.34	27.86 ± 5.08	25.28 ± 3.03

TC (mmol/L)	3	2.10 ± 0.07	1.94 ± 0.23	2.15 ± 0.09	2.01 ± 0.26
	10	1.68 ± 0.13	4.95 ± 1.24*	4.25 ± 1.29*	4.27 ± 1.31*
	15	1.87 ± 0.28	5.49 ± 1.47*	4.11 ± 1.14^∗▲^	3.78 ± 0.98^∗▲^

TG (mmol/L)	3	0.57 ± 0.13	0.18 ± 0.03	0.33 ± 0.01	0.15 ± 0.02
	10	0.74 ± 0.13	5.26 ± 1.76*	1.63 ± 0.59^▲^	1.43 ± 0.41^▲^
	15	0.39 ± 0.14	2.24 ± 1.44*	0.60 ± 0.36^▲^	0.51 ± 0.21^▲^

HDL (mmol/L)	3	1.54 ± 0.23	1.63 ± 0.21	2.16 ± 0.10	1.75 ± 0.19
	10	1.41 ± 0.1	3.52 ± 0.63*	3.37 ± 0.65*	2.92 ± 0.62*
	15	1.52 ± 0.15	4.06 ± 0.7*	3.19 ± 0.74^▲^	3.0 ± 0.62^▲^

LDL (mmol/L)	3	0.84 ± 0.14	0.89 ± 0.13	1.1 ± 0.12	0.9 ± 0.10
	10	0.63 ± 0.11	2.38 ± 0.67*	2.51 ± 068*	1.98 ± 0.48*
	15	0.84 ± 0.12	2.18 ± 0.83*	1.77 ± 0.63^∗▲^	1.6 ± 0.37^∗▲^

There were significant decrease of TP, Alb, and elevation of TC, TG, HDL, and LDL levels of the PAN group compared with that of the sham group on days 10 and 15 (*P* < 0.05). YQQRG could significantly reduce TG at day 10. At day 15, YQQRG increased plasma levels of TP and Alb and reduced TC, TG, and LDL levels (*P < 0.05 compared with sham group; ^▲^ *P* < 0.05 compared with PAN). (ALT: alanine transarninase; AST: aspartate aminotransferase; Cre: creatinine; BUN: blood urea nitrogen; TP: total protein; Alb: albumin; TC: total cholesterol; TG: triglycerides; HDL: high density lipoprotein; and LDL: low density lipoprotein.)
